# Bone health in functional hypothalamic amenorrhea: What the endocrinologist needs to know

**DOI:** 10.3389/fendo.2022.946695

**Published:** 2022-10-11

**Authors:** Rita Indirli, Valeria Lanzi, Giovanna Mantovani, Maura Arosio, Emanuele Ferrante

**Affiliations:** ^1^ Department of Clinical Sciences and Community Health, University of Milan, Milan, Italy; ^2^ Endocrinology Unit, Fondazione Istituto di Ricovero e Cura a Carattere Scientifico (IRCCS) Ca’ Granda Ospedale Maggiore Policlinico, Milan, Italy

**Keywords:** functional hypothalamic amenorrhea (FHA), female athlete triad, bone, osteoporosis, oral contraceptives (OCs), estrogen, anorexia nervosa

## Abstract

In the original definition by Klinefelter, Albright and Griswold, the expression “hypothalamic hypoestrogenism” was used to describe functional hypothalamic amenorrhoea (FHA). Given the well-known effects of estrogens on bone, the physiopathology of skeletal fragility in this condition may appear self-explanatory. Actually, a growing body of evidence has clarified that estrogens are only part of the story. FHA occurs in eating disorders, overtraining, and during psychological or physical stress. Despite some specific characteristics which differentiate these conditions, relative energy deficiency is a common trigger that initiates the metabolic and endocrine derangements contributing to bone loss. Conversely, data on the impact of amenorrhoea on bone density or microarchitecture are controversial, and reduced bone mass is observed even in patients with preserved menstrual cycle. Consistently, oral estrogen-progestin combinations have not proven beneficial on bone density of amenorrheic women. Low bone density is a highly prevalent finding in these patients and entails an increased risk of stress or fragility fractures, and failure to achieve peak bone mass and target height in young girls. Pharmacological treatments have been studied, including androgens, insulin-like growth factor-1, bisphosphonates, denosumab, teriparatide, leptin, but none of them is currently approved for use in FHA. A timely screening for bone complications and a multidisciplinary, customized approach aiming to restore energy balance, ensure adequate protein, calcium and vitamin D intake, and reverse the detrimental metabolic-endocrine changes typical of this condition, should be the preferred approach until further studies are available.

## Introduction

Functional hypothalamic amenorrhea (FHA) is a condition of chronic hypoestrogenism without identifiable organic causes (1). It is encountered in undernutrition and eating disorders (e.g. anorexia nervosa, AN), overtraining, emotional stress and chronic diseases (2), and entails long-term consequences, including bone loss (1).

The definition of osteopenia and osteoporosis in AN is inconsistent among studies: while some have considered bone mineral density (BMD) T-scores (osteopenia: -1.0<T-score<-2.5; osteoporosis: T-score<-2.5), others have reported z-scores and defined osteopenia at -1.0<z-score<-2.0 and osteoporosis at z-score<-2.0. Overall, osteopenia is reported in 25-90% and osteoporosis in 19-44% of adult women with AN (3–6). More than a half of AN adolescent girls present z-score<-1 at one or more sites, most commonly the spine (7). In amenorrheic AN women, BMD declines by 2.4% at the hip and by 2.6% at the spine annually (8).

Impaired microarchitecture (9–13) and bone strength (9) have been documented in AN, resulting in a cumulative incidence of fragility fractures up to 57% (14). Fracture risk is increased at all ages and at several sites, particularly the hip, pelvis, spine and distal forearm (15, 16).

Women with exercise-related FHA have low BMD, even though to a lesser extent than AN patients (17). The “Female Athlete Triad” is a condition characterized by low energy availability, FHA, and osteoporosis (18). Z score<-2.0 and -1.0<z-score<-2.0 have been reported in 0-15.4% and 0-39.8% of female athletes respectively (19). This variability may result from the varied effects which different sports exert on bone (20). Amenorrheic athletes also have impaired microarchitecture (21, 22) and bone strength (22), and a higher risk of stress fractures (28-47%) compared to eumenorrheic athletes (17-25.6%) and nonathletes (23, 24).

When FHA manifests at young age, it irreversibly impairs bone mass accrual, since 90% of peak bone mass (PBM) is achieved by the age of 18 (25). Adult women with AN onset before age 18 show lower spine BMD than those developing it later, regardless of amenorrhea duration (26). Additionally, final height can be impaired (27) and bone maturation delayed (28). Despite weight and menstrual recovery, individuals who experience bone loss as adolescents have chronic deficits and an increased risk of fracture in adulthood (29–31).

In this review, we summarize determinants of bone loss, pitfalls in assessment and treatment, and indications for management of FHA-related skeletal fragility.

## Determinants of bone loss

As a condition of estrogen deficiency, skeletal involvement may appear straightforward in FHA, since estrogens exert a predominantly antiresorptive action on bone (32) and, along with growth hormone (GH), insulin-like growth factor 1 (IGF-1) and energy balance, play a key role in pubertal growth and bone mass accrual (33).

### Hypoestrogenism

Delayed menarche and longer amenorrhea duration are associated with low BMD, altered microarchitecture, reduced strength, and fractures in AN- (5, 26, 34), stress- (17) and exercise-FHA (22, 30, 35, 36). Age of onset is critical since estrogen deficiency during adolescence determines low PBM, which adds to hypoestrogenism-related bone loss during adulthood (26). However, the estradiol threshold considered to have skeletal effects in the general female population has classically been set at 25-30 pg/ml and further lowered to 5 pg/ml in subsequent studies (37), while serum concentrations are generally higher in FHA (17, 21). Additionally, reduced BMD is found in eumenorrheic AN women too, particularly at the hip (38). These observations suggest that hypoestrogenemia is not the only determinant of bone loss, and effects may vary at different skeletal sites. Indeed, Miller et al. documented low BMD at the spine, hip and radius in AN women, and only at the spine in normal-weight women with other forms of FHA (39). Spine BMD is significantly lower in amenorrheic athletes than in eumenorrheic ones (24, 35), while femoral BMD is comparable (35). Therefore, hypoestrogenism appears to impact mainly on trabecular (e.g. spine) bone, while other factors like body mass index, lean mass and mechanical load act on cortical (e.g. hip) bone (8, 34, 35, 38).

### Lifestyle factors

Energy imbalance is the initial trigger for the (mal-)adaptive changes and comorbidities observed in FHA (40, 41).

**Figure 1:**
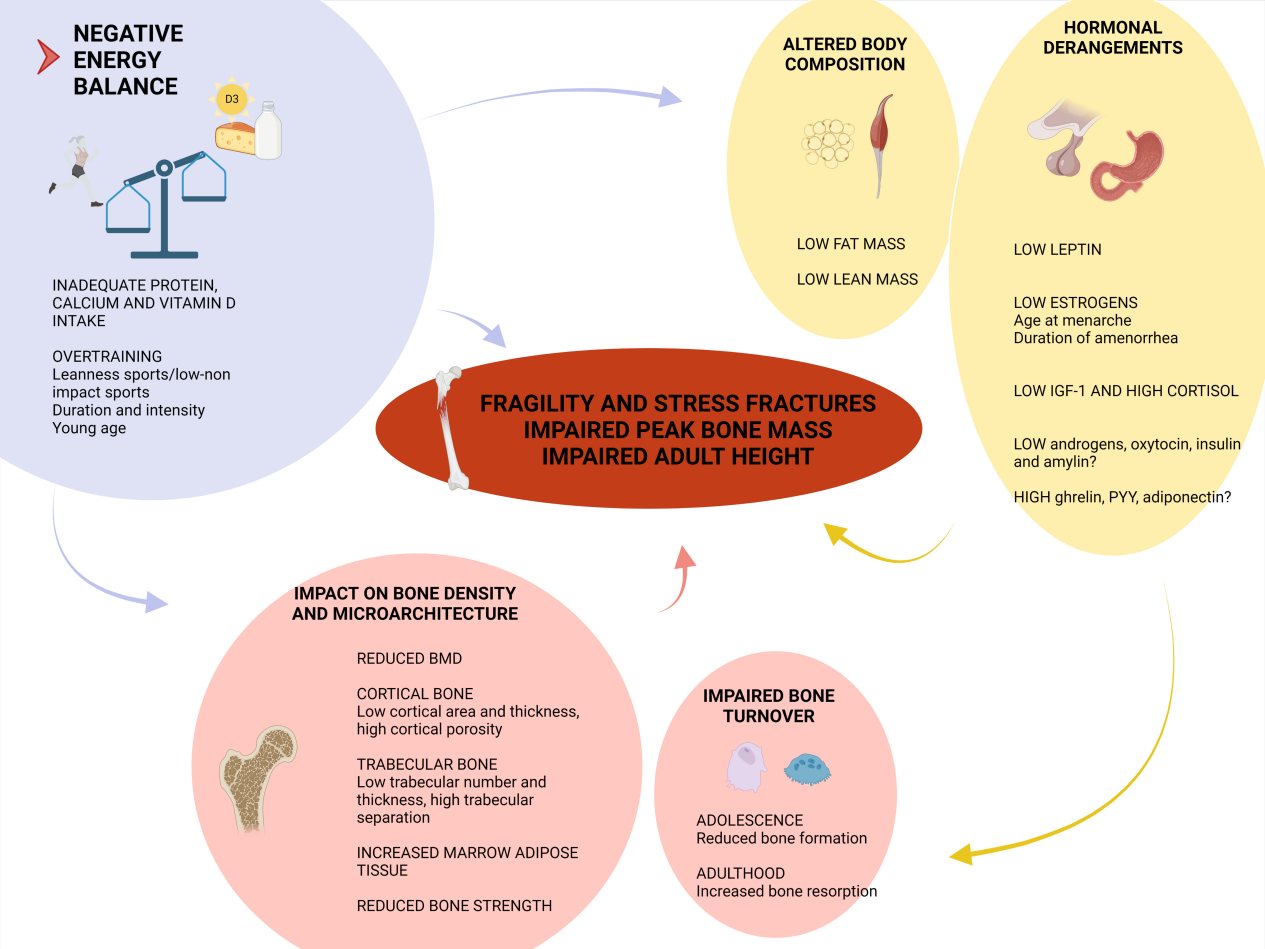
Determinants of skeletal fragility in functional hypothalamic amenorrhea (FHA). Negative energy balance is the prime determinant in FHA physiopathology. Not only the caloric intake, but also the insufficient amount of dietary calcium, vitamin D and proteins impact on bone. In case of overtraining, some features of sports, like mechanical load and exercise intensity, can affect bone health. The reduction in lean mass impairs peak bone mass achievement and cortical bone microarchitecture. The reduction in fat mass is associated with low leptin levels and hypogonadism. Estrogen deficiency contributes to the increased bone resorption (mainly observed in adulthood) and altered trabecular bone mineral density (BMD) and microarchitecture. The low levels of insulin-like growth factor-1 (IGF-1) result from growth hormone resistance and the nutritional deprivation, and participate in lowering bone turnover as observed in adolescent patients, and in disrupting peak bone mass achievement. The hypothalamus-pituitary-adrenal axis is overactive in FHA, resulting in enhanced cortisol secretion which, in turn, inhibits intestinal calcium absorption, increases urinary calcium excretion, inhibits osteoblast proliferation and increases marrow fat content. Further studies are needed to clarify whether testosterone, dehydroepiandrosterone, ghrelin, peptide YY (PYY), adiponectin, insulin, amylin and oxytocin play a role in FHA skeletal involvement (42, 43). (Created with BioRender.com).

Energy deficit yields changes in hypothalamus-pituitary axes, adipokines and gastrointestinal hormones which, in turn, affect bone (42, 43). Describing this neuro-endocrine adaptation is beyond the purpose of this review. However, in order to understand the rationale behind treatment approaches, the lifestyle and the hormonal contributors to bone disease are summarized in **Figure 1** (42, 43).

Calcium and vitamin D intake, physical activity and lean body mass influence PBM (25). Untreated AN patients present lower circulating levels of 25OH-vitamin D and 1,25OH-vitamin D than controls (44). Vitamin D insufficiency (serum 25OH-vitamin D<30 ng/mL) is observed in more than 50% AN women and is associated with higher parathyroid hormone concentrations and lower hip BMD (45). Among adolescent female gymnasts, 83.3% present vitamin D insufficiency, 33.3% vitamin D deficiency (<20 ng/mL), and 72.2% a poor dietary calcium intake (46), and these factors are associated with impaired bone turnover (47).

The impact of exercise on bone is complex, since the entity of mechanical loading (48) and exercise intensity (49) influence bone metabolism and fractures and interact with energy and gonadal status. Activities with high or odd mechanical strain, like ball, power or antigravitational sports, induce bone mass gain and improve bone geometry and strength (20, 21, 50), particularly at weight-bearing sites. Conversely, in sports generating low or repetitive loading, like endurance running, ballet and swimming, the detrimental effects of energy deficit prevail (20, 51).

The effects of exercise in AN are controversial and vary according to exercise intensity, mechanical loading, and phase of illness. High bone-loading activities performed for 1-6 hours/week during recovery from AN, may enhance bone accrual. Conversely, low-mechanical loading activities performed for <1 or >6 hours/week increase risk of bone loss (52, 53).

### Chronic diseases

Some conditions associated with FHA (54) -like HIV infection, organ transplant- can cause bone loss *per se*, and/or because of medications used (antiretrovirals, glucocorticoids) (55). Also some drugs, like some antidepressants, can directly contribute to both FHA and osteoporosis (55).

**Table 1** summarizes determinants of bone loss in different FHA forms.

**Table 1:** Determinants of bone loss in different forms of functional hypothalamic amenorrhea.

Undernutrition / Eating disorders	Overtraining	Systemic diseases / psychological stress
Delayed menarche, amenorrhea Young age with impaired peak bone mass Inadequate calorie, protein and calcium intake Vitamin D insufficiency/deficiency Hyponatremia Low mechanical-loading physical activity (e.g. running) Low body weight with low fat mass and low lean mass Drugs: Diuretics abuse Selective serotonin re-uptake inhibitors Endocrine modifications:↓Leptin↓GnRH pulsatility, LH, FSH and estrogens↑CRH, ACTH and cortisol↑GH with GH resistance, ↓IGF-1 ↓Androgens ↓Oxytocin, insulin, amylin ? ↑PYY, ghrelin, adiponectin ?	Delayed menarche, amenorrhea Young age with impaired peak bone mass Relative energy deficiency Inadequate protein or calcium intake Vitamin D insufficiency/deficiency Possible coexistence of eating disorders Sports with low or repetitive loading (e.g. endurance running, ballet, swimming) Leanness sports Training intensity Low body weight with low fat mass Endocrine modifications: ↓Leptin ↓GnRH pulsatility, LH, FSH and estrogens ↑CRH, ACTH and cortisol ↓IGF-1 ↑Ghrelin ↑PYY ↑ or = adiponectin ↓Insulin ↓Oxytocin	Delayed menarche, amenorrhea Young age with impaired peak bone mass Underlying conditions and/or drugs: Gastrointestinal malabsorption (inflammatory bowel disease, coeliac disease) Chronic inflammatory diseases with hypermetabolic states (e.g. rheumatoid arthritis) Organ failure (e.g. cystic fibrosis, chronic renal disease, liver disease) Organ transplant and medications (e.g. glucocorticoids, immunosuppressants) HIV infection and medications Diabetes (types 1 and 2) Depression and/or selective serotonin re-uptake inhibitors Endocrine modifications: ↓GnRH pulsatility, LH, FSH and estrogens ↑CRH, ACTH and cortisol Immune stress (IL-1β, IL-6, TNF-α)

GnRH, Gonadotropin-releasing hormone. LH, luteinizing hormone. FSH, follicle-stimulating hormone. CRH, corticotropin-releasing hormone. ACTH, adrenocorticotropic hormone. Up arrow, increase. Down arrow, reduction. =, no variation. GH, growth hormone. IGF-1, insulin-like growth factor 1. PYY, peptide YY. IL-1β, interleukin 1β. IL-6, interleukin 6. TNF-α, tumor necrosis factor α.

## Skeletal evaluation

### Bone density

Dual-energy X-ray absorptiometry (DXA) is used for evaluation of areal BMD. However, heterogeneous definitions of low BMD in FHA have been used in research studies and by scientific societies.

According to the International Society for Clinical Densitometry (56), z-score rather than T-score should be considered in pre-menopausal women, for whom there are no densitometric criteria of osteopenia and osteoporosis. Instead, BMD is defined as “below the expected range for (chronological) age” if z-score is <-2 (57, 58). In adult women, a diagnosis of osteoporosis is established if secondary causes of low BMD or risk factors for fracture are present too (57, 58), while in adolescents a clinically significant fracture history is required (59, 60).

The definition of low BMD in the 2007 position statement of the American College of Sports Medicine, is substantially different: low BMD in premenopausal athletes is defined at -2<z-score<-1 (18), considering that sportswomen have 5–15% higher BMD than nonathletes. More recently, the Female Athlete Triad Coalition and the Endocrine Society differentiated weight-bearing and non-weight-bearing sports (1, 61). In the former case -1.0<z-score<-2.0 deserves attention; for other sports, low BMD is diagnosed at z-score<-2.

Physicians should bear in mind that z-score>−2.0 does not exclude skeletal fragility. In fact, BMD explains 60-80% of bone strength and does not encompass other skeletal features (62).

### Bone quality

Trabecular bone score is a textural index that provides an indirect measurement of lumbar spine trabecular microarchitecture (63, 64). Trabecular bone score is impaired in a significant percentage of AN adolescents and may represent a useful tool for skeletal evaluation (64, 65).

High-resolution peripheral quantitative computed tomography allows characterization of volumetric BMD, bone geometry and microarchitecture. Studies with this technique documented decreased cortical area and thickness, higher cortical porosity, lower trabecular number and thickness, and increased trabecular separation, in AN patients (9, 11, 66) and in amenorrheic athletes (21–23). Patients with multiple fractures have the most significant microarchitecture deterioration (23).

### Morphometric vertebral fractures

The prevalence of asymptomatic vertebral fractures in FHA is unknown. One study including 80 young AN women found a low rate of prevalent and incident morphometric fractures, which were not predicted by BMD, duration or severity of malnutrition (67). While the screening of asymptomatic vertebral fractures is recommended in primary and most secondary forms of osteoporosis (55), there is no such indication in FHA.

### Biochemical markers

Bone formation and resorption markers are used as indicators of treatment efficacy and compliance (55). Although they are not indicated for routine patients’ evaluation (55), they can help characterize the turnover status of an individual.

Energy and estrogen status affect turnover in a time-dependent manner: while adolescents with AN show mainly reduced formation (68), in adult women enhanced bone resorption prevails (69), resulting in uncoupling of bone metabolism.

Assessment of estradiol levels has poor diagnostic significance since menstrual periods reflect estrogen status. However, current guidelines suggest to collect this value (1), which may serve for the differential diagnosis with polycystic ovary syndrome (70) and the therapeutic decision-making in patients planning pregnancy (1). Conversely, usefulness in the diagnostic and therapeutic work-up of skeletal complications is not defined and is probably limited.

## Lifestyle intervention

Evidence on lifestyle and pharmacological approaches is summarized in **Table 2**.

**Table 2:** Summary of evidence of lifestyle change and pharmacologic treatments.

Lifestyle change and pharmacologic treatment	Reference	Clinical evidence
**Weight gain**	Giollo et al. (71)	Increase in spine BMD by 1.1% over 20 weeks; no change in hip
	Mika et al. (72)	No change over 2 years
	Compston et al. (73); Gordon et al. (74)	No change over 1 year
	Viapiana et al. (75)	Increase in spine and hip BMD by 4.8 and 7.1% respectively over 15 months
**Weight gain + menses restoration**	Miller et al. (8)	Mean annual increase in spine and hip BMD by 3.1 and 1.8% respectively
	Misra et al. (76)	Stabilization of BMD measures over 9 months
	Dominguez et al. (77)	Increase in spine and hip BMD by 4.6 and 3.1% respectively over 2.2 months
**Calcium and vitD supplementation**	**-**	**-**
**Oral contraceptives**	Grinspoon et al. (78); Vescovi et al. (79)	Reduction in bone resorption and formation markers
	Golden et al. (80); Strokosch et al. (81)	No increase in BMD over 1 year vs placebo
	Warren et al. (82); Hergenroeder et al. (83)	Improvement in lumbar spine but not hip BMD over 1 year vs placebo
	Sowińska et al. (84)	Increase in BMD from baseline over 4 years
**Transdermal estradiol**	Misra et al. (85); Ackerman et al. (86)	Increase in spine and hip BMD over 18 months vs placebo
**Androgens**	Miller et al. (87)	No changes in osteocalcin and BALP levels after transdermal testosterone vs placebo
	Miller et al. (88)	No increase in BMD from baseline over 12 months transdermal testosterone
	Bloch et al. (89)	No increase in BMD after DHEA vs placebo
	Di Vasta et al. (90)	Stabilization of femoral neck BMD after DHEA + oral contraceptives vs placebo
**IGF-1**	Misra et al. (91); Grinspoon et al. (92)	Increase in bone formation markers vs placebo
	Grinspoon et al. (93)	Improvement in BMD after IGF-1 + oral contraceptives vs IGF-1 alone
	Fazeli et al. (94)	No changes in bone turnover markers after rh-GH
**Bisphosphonates**	Golden et al. (95)	Increase in femoral neck but not lumbar spine BMD after alendronate
	Miller et al. (96); Miller et al. (88)	Increase in spine BMD after risedronate
	Haines et al. (97)	Increase in spine BMD after risedronate + IGF-1 vs risedronate alone
**Denosumab**	Jamieson et al. (98)	Increase in spine, hip and femoral neck BMD
**Teriparatide**	Milos et al. (99); Shibli-Rahhal et al. (100)	Increase in femoral neck BMD
	Fazeli et al. (101)	Increase in lumbar spine BMD
**Recombinant leptin**	Welt et al. (102); Chou et al. (103); Foo et al. (104)	Increase in bone formation markers
	Sienkiewicz et al. (105)	Increase in lumbar spine BMD

BMD, bone mineral density. vitD, vitamin D. BALP, bone alkaline phosphatase. DHEA, dehydroepiandrosterone. IGF-1, recombinant human insulin-like growth factor 1. rh-GH, recombinant human Growth Hormone.

Correction of energy deficit, weight recovery and resumption of menses are primary goals, and the finding of low BMD may motivate patients towards behavioural changes (106). Positive energy balance can be achieved by reducing exercise energy expenditure and/or increasing caloric intake, according to published recommendations (107). However, a threshold level of weight or body mass index gain is not established (76). An experienced multidisciplinary team is advocated for the management of these patients, including nutritionist, psychologist or psychiatrist, athletic trainer, internist, sports physician (106).

Weight gain reverses uncoupling of bone remodelling by increasing bone formation and reducing bone resorption in the short- and middle-term (108, 109). Weight improvement and resumption of menses are associated with BMD stabilization or increase (8, 72–75), even if some studies reported conflicting results (77, 110, 111). A longitudinal study demonstrated normalisation of spine BMD, bone volume and volumetric BMD in adolescents 2.7 years after recovery from AN (112). However, some observations suggested that only a partial effect can be achieved with weight recovery alone without restoration of gonadal function (71, 75, 113), or at least that a differential effect is exerted by these two factors: in a study by Miller et al., BMD increased at the hip following weight gain, and at the spine following menstrual recovery (8).

An adequate intake of calcium and vitamin D is generally recommended to ensure bone health (55). No study has specifically addressed this issue in FHA (76, 78). However, hypovitaminosis D may counteract the efficacy of refeeding in AN (79). Therefore, recommendations on adequate calcium and vitamin D intake appear appropriate (76).

However, bone disease may be not completely reversible, as low BMD and increased fracture risk can persist lifelong after sustained recovery from FHA (14, 75, 82).

## Pharmacological treatment

### Estrogens

Trials with oral contraceptives (OCs) or hormonal replacement therapy have led to controversial results. Reduction in bone resorption but also formation markers has been reported with OCs in FHA (80, 81, 83). In AN women, OCs did not increase BMD significantly over 1 year compared with no treatment or placebo (84, 85), and osteopenia persisted or progressed after 3 years (84). Conversely, in women with other forms of FHA, two placebo-controlled trials reported improvement in spine, but not hip BMD after 1 year (83, 86). A 4-year sequential therapy with 17β-estradiol and dydrogesterone significantly increased BMD from baseline (87). According to other findings, OCs may even be detrimental for bone mass recovery: in an observational study, AN women receiving OCs showed no BMD improvement despite weight gain, whereas hip BMD increased in women who gained weight but did not receive OCs (8).

On the other side, hormonal replacement therapy (i.e. transdermal estradiol with cyclic progesterone) over 12-18 months yielded a significant increase in spine and hip BMD in adolescents and young women with FHA compared with placebo (88) or OCs (89).

The controversial efficacy of OCs, as opposed to the transdermal estrogen administration, is ascribed to the further lowering of IGF-1 and (free) androgens concentrations caused by the former (39) (**Figure 1**). In addition, the ethinyl-estradiol content of OCs has been progressively reduced to the minimum effective dose, because of concerns on thromboembolic events. Subsequently, conflicting results have been reported about the effects on bone of low-dose and very-low-dose OCs in young women (90–92).

### Androgens

Two studies reported on the use of low-dose transdermal testosterone in AN. The first failed to find significant changes in bone formation markers versus no treatment (93) and the second did not document BMD improvement from baseline following 12-month therapy (94).

In AN women, two placebo-controlled trials found no increase in BMD at any site with dehydroepiandrosterone alone (95), and a stabilization of femoral neck BMD with dehydroepiandrosterone given in combination with an OC for 18 months (96).

### IGF-1

Short-term therapy with recombinant human IGF-1 increases bone formation markers in AN patients compared to placebo (97, 98). Improvement in BMD is observed when IGF-1 is given in combination with OCs (101). Supraphysiological recombinant human GH administration does not affect turnover markers in AN women (99).

### Bisphosphonates

Femoral neck, but not spine BMD increased from baseline after 1 year of treatment with alendronate (100), while risedronate improved spine BMD compared to placebo when administered for 1 year in AN women (94, 102). A sequential therapy with IGF-1 followed by risedronate increased spine BMD more than risedronate alone (103).

### Denosumab

Experience with denosumab is anecdotal. Increase in spine and hip BMD has been reported in a woman with AN treated with denosumab for 3 years (104).

### Teriparatide

Three studies reported a positive effect of teriparatide on spine BMD after 6 months (105) and on femoral neck BMD after 2 years (114, 115) compared to placebo in women with AN and severe osteoporosis.

### Recombinant leptin

Leptin administration for 3-9 months in women with FHA improves gonadal, thyroid and growth hormone axes function, bone formation markers, RANK-ligand/osteoprotegerin ratio (116–118), and spine BMD after 2 years compared with no treatment (119). However, weight loss was observed in leptin-treated patients (119), making this treatment unsuitable for low-weight women with FHA.

## Discussion

FHA is responsible for 20-35% cases of secondary amenorrhea (120). Some forms of FHA can take a long time to recover (121), and all bring about long-term health consequences (120). In addition, FHA involves a wide range of hormonal changes which differentiate it from other conditions of estrogen deficiency and make its management challenging.

The first step in the work-up of FHA-related bone loss, consists in selecting patients for clinical evaluation. Current guidelines recommend to screen patients with FHA lasting ≥6 months, or earlier if other risk factors occur, including low body weight, eating disorders, delayed menarche or prior fractures (1, 61). DXA scan of lumbar spine, hip and, in adolescents, whole body, is recommended for BMD evaluation, while parameters like trabecular bone score and bone microarchitecture should be reserved to research purposes. Only z-score has to be considered in pre-menopausal women, and low BMD is defined as z-score<-2 (1, 61). DXA scan has to be repeated every 12-24 months in patients at risk for bone loss and to monitor treatment (1, 18, 61). As no recommendation exists about screening for asymptomatic vertebral fractures, we suggest that the decision is left to clinical judgement, according to the presence of other risk factors or comorbidities contributing to skeletal demineralization (55).

Studies on pharmacological treatments have led to no definitive conclusions. Level of evidence is low, as based on few randomized controlled trials of short duration, or observational studies including small cohorts. Moreover, effects have been evaluated in terms of BMD or turnover markers, while data on fracture prevention are lacking.

Based on FHA-related hormonal changes, treatments with estrogens, IGF-1, leptin, androgens have been attempted. However, one single hormonal therapy has no or little effect on the other mechanisms which influence bone metabolism.

Administration of OCs has led to conflicting results. In addition, physicians should consider that resumption of menses through estrogen prescription could reduce patient’s motivation to gain weight (122). Nevertheless, it is estimated that up to 78% of physicians prescribe OCs inappropriately to prevent bone loss (122).

Bone active medications are promising alternatives but more data are needed before including them in treatment recommendations. Bone active drugs should be prescribed cautiously in young women of reproductive age, considering that they cannot be administered for long periods, some of them raise concerns about fracture risk after discontinuation (123, 124), and the long half-life of bisphosphonates.

Both the Female Athlete Triad Coalition and the Endocrine Society suggest prescription of transdermal estradiol with cyclic progestins (but not OCs) in high-risk patients (i.e. z-score<-2.0, prior fractures) who did not respond to 1 year of non-pharmacological therapy (i.e. further BMD loss or new fracture) (1, 61). While the Endocrine Society recommends against the use of other medications (1), the Coalition suggests to consider bone active agents in women with contraindications to or lack of benefit from estrogen replacement (61).

At present, the cornerstone of management of FHA-related bone loss is lifestyle intervention. Weight gain has the most robust impact on BMD, and recovery of gonadal function has an additive effect (125). Patients should be aware that normalization of energy balance is the main factor that anticipates resumption of menses and BMD gain; however, achieving these goals takes months or years. Regular monitoring and long-lasting support should be provided by an experienced multidisciplinary team (18).

Nutritional intervention should address caloric intake and micro- and macronutrients availability. Since no data are available on the optimal daily intake of calcium and vitamin D in FHA, recommendations for other forms of osteoporosis are adopted (55, 78), i.e. 1000–1300 mg of calcium and 400–800 IU of vitamin D daily, eventually through oral supplements (18, 61). Serum 25OH-vitamin D levels should be monitored, aiming at concentrations of 30-50 ng/ml. Protein intake should be customized considering that intensely training athletes have higher requirements (18).

Energy availability can be increased by reducing exercising intensity too. This should be planned with a sports physician and athletic trainer for female athletes. In AN, moderate physical activity may be acceptable during the recovery phase (52, 53).

Lifestyle intervention must continue also in patients who receive pharmacological treatments.

In conclusion, FHA is a condition of estrogen deficiency which entails several metabolic and hormonal alterations. Bone is severely affected, with long-term consequences including short stature, reduced PBM and skeletal fragility. While re-establishing adequate energy balance and nutrients intake is instrumental to weight gain, recovery of menses and resolution of hormonal derangements, bone impairment is not completely reversible and the increased fracture risk can persist life-long. Preventive educational programs should be undertaken in schools or during athletic training. Larger and longer-lasting trials, new therapeutic approaches and combined strategies are warranted to improve bone health.

## Author contributions

RI, VL, EF searched and selected the scientific literature and prepared the manuscript. GM and MA critically revised the manuscript. All authors approved the submitted version and agree to be accountable for the content of the work.

## Funding

This work was funded by University of Milan, Milan, Italy.

## Conflict of interest

The authors declare that the research was conducted in the absence of any commercial or financial relationships that could be construed as a potential conflict of interest.

## Publisher’s note

All claims expressed in this article are solely those of the authors and do not necessarily represent those of their affiliated organizations, or those of the publisher, the editors and the reviewers. Any product that may be evaluated in this article, or claim that may be made by its manufacturer, is not guaranteed or endorsed by the publisher.
